# Ellagic Acid Attenuates CCl_4_-Induced Hepatic Fibrosis and Is Associated with Changes in PI3K/AKT- and EMT-Related Protein Expression

**DOI:** 10.3390/cimb48070711

**Published:** 2026-07-13

**Authors:** Di Tan, Mengyi Qiao, Qingqing Zhang, Chuan Wang, Xue Bai, Fang Peng, Dingyu Wu

**Affiliations:** Yunnan Key Laboratory of Insect Biomedical R&D, School of Pharmacy, Dali University, Dali 671000, China; tandi@dali.edu.cn (D.T.); qiaomengyi0319@163.com (M.Q.); zhangqingqing1010@163.com (Q.Z.); wangchuan66202009@163.com (C.W.); 18387795812@163.com (X.B.); pengfang6556@aliyun.com (F.P.)

**Keywords:** ellagic acid, hepatic fibrosis, epithelial–mesenchymal transition, PI3K/AKT signaling, network pharmacology

## Abstract

Hepatic fibrosis is characterized by excessive extracellular matrix deposition and progressive impairment of liver structure and function. Ellagic acid (EA) has been reported to have antioxidant and anti-inflammatory properties, but its role in hepatic fibrosis remains incompletely understood. A CCl_4_-induced mouse model of hepatic fibrosis was established. EA was administered by oral gavage at 30, 60, or 120 mg/kg; colchicine was used as a positive control. Histopathological changes and collagen deposition were assessed using hematoxylin and eosin and Sirius Red staining, respectively. Serum alanine aminotransferase (ALT), aspartate aminotransferase (AST), interleukin-6 (IL-6), and interleukin-10 (IL-10) concentrations, as well as hepatic malondialdehyde (MDA) and hydroxyproline (Hyp) contents and total superoxide dismutase (T-SOD) activity, were determined. Immunohistochemistry and Western blotting were used to assess fibrosis-associated, EMT-associated, and PI3K/AKT-related proteins. Network pharmacology analysis was performed to identify candidate targets and pathways. EA treatment ameliorated histopathological injury and collagen deposition in CCl_4_-treated mice. EA, particularly at the high dose, reduced serum ALT and AST activities and hepatic MDA and Hyp contents while increasing hepatic T-SOD activity. EA treatment was also associated with lower serum IL-6 and higher IL-10 concentrations. In the EA-H group, α-SMA and collagen I (COL1A1) immunoreactivity were reduced; the abundance of N-cadherin, PI3K, and AKT was lower; and E-cadherin expression was higher. Network pharmacology identified PI3K/AKT-related signaling as a candidate pathway associated with the anti-fibrotic effects of EA. EA attenuated CCl_4_-induced hepatic fibrosis and was associated with lower oxidative stress, inflammation, and HSC activation. Direct PI3K/AKT inhibition was not established.

## 1. Introduction

Hepatic fibrosis (HF) is a common pathological consequence of chronic liver diseases of diverse etiologies. It is characterized by hepatic stellate cell (HSC) activation and excessive extracellular matrix (ECM) accumulation and may progress to cirrhosis and hepatocellular carcinoma, thereby imposing a substantial global health burden [1]. EMT-associated molecular changes have received considerable attention in fibrogenesis because they are linked to phenotypic reprogramming of hepatocytes and biliary epithelial cells toward mesenchymal-like states. Such changes may contribute to ECM production and deposition. Activated HSCs may secrete bone morphogenetic protein 1 (BMP-1), which has been reported to induce EMT-associated changes in hepatocytes, including loss of epithelial characteristics, acquisition of mesenchymal features, and increased ECM accumulation [2].

The phosphoinositide 3-kinase/protein kinase B (PI3K/AKT) signaling pathway plays an important role in regulating cell proliferation, survival, and EMT-associated molecular changes. Aberrant activation of this pathway has been reported to promote HSC activation and collagen synthesis, contributing to fibrotic progression [3,4]. Increasing evidence indicates that PI3K/AKT signaling interacts with inflammatory pathways, including nuclear factor κB (NF-κB), to regulate fibrogenic responses [5]. Accordingly, modulation of PI3K/AKT-related signaling, EMT-associated changes, and inflammatory responses may represent a potential therapeutic strategy for HF.

Ellagic acid (EA) is a naturally occurring polyphenolic compound found in pomegranate, berries, and some medicinal plants. It has been reported to exert antioxidant, anti-inflammatory, and anti-fibrotic effects [6]. Previous studies have shown that EA can attenuate oxidative stress, reduce inflammatory cytokine production, and ameliorate histopathological liver injury in experimental models, including non-alcoholic fatty liver disease (NAFLD), suggesting potential hepatoprotective effects during fibrotic progression [7]. In a renal fibrosis model, EA attenuated EMT-associated protein changes [8]. Nevertheless, the molecular mechanisms underlying the anti-fibrotic effects of EA in the liver remain incompletely understood. In particular, it remains unclear whether EA attenuates HSC activation and ECM deposition in association with PI3K/AKT-related signaling and EMT-associated protein alterations during hepatic fibrosis. The potential multi-target pharmacological actions of EA and its applicability across different etiologies of hepatic fibrosis also require further investigation.

In the present study, network pharmacology was integrated with in vivo experimental validation to identify putative targets and signaling pathways associated with the anti-fibrotic effects of EA. Particular attention was given to PI3K/AKT-related signaling and EMT-associated protein alterations. This study aimed to provide experimental and network-based evidence for the protective effects of EA against hepatic fibrosis and to inform future studies of natural polyphenol-based therapeutic strategies for liver fibrosis.

## 2. Materials and Methods

### 2.1. Animals

Forty-two SPF-grade male C57BL/6J mice, aged 8 weeks and weighing 20–22 g, were purchased from Speifu (Beijing) Biotechnology Co., Ltd. (Beijing, China; production license no. SCXK (Beijing) 2024-0001).

### 2.2. Reagents and Antibodies

Ellagic acid (purity ≥ 98%; cat. no. E808704) was obtained from Shanghai Macklin Biochemical Technology Co., Ltd. (Shanghai, China). Colchicine was purchased from Sinopharm Chemical Reagent Co., Ltd. (Shanghai, China). Carbon tetrachloride (CCl_4_; analytical grade; cat. no. HY-Y0298) was obtained from MedChemExpress LLC (Monmouth Junction, NJ, USA). Methanol (analytical grade; product no. 231221) was obtained from Sichuan Xilong Scientific Co., Ltd. (Chengdu, China).

The primary antibodies used in this study included antibodies against AKT (cat. no. 10176-2-AP; Proteintech Group, Inc., Rosemont, IL, USA), PI3K p110α (cat. no. A22730; ABclonal Technology Co., Ltd., Wuhan, China), α-SMA (cat. no. A2235; ABclonal Technology Co., Ltd., Wuhan, China), COL1A1 (cat. no. A24112; ABclonal Technology Co., Ltd., Wuhan, China), E-cadherin (cat. no. GB11868-100; Wuhan Servicebio Technology Co., Ltd., Wuhan, China), N-cadherin (cat. no. GB111273-100; Wuhan Servicebio Technology Co., Ltd., Wuhan, China), and vimentin (cat. no. GB11192-100; Wuhan Servicebio Technology Co., Ltd., Wuhan, China). HRP-conjugated goat anti-rabbit IgG (cat. no. SA0001-2; Proteintech Group, Inc., Rosemont, IL, USA) was used as the secondary antibody.

The BCA Protein Quantification Kit (cat. no. E112) was purchased from Vazyme Biotech Co., Ltd. (Nanjing, China). The Malondialdehyde (MDA) Assay Kit for tissue and blood samples (cat. no. G4302) and the Total Superoxide Dismutase (T-SOD) Activity Assay Kit (cat. no. G4306-48T) were purchased from Wuhan Servicebio Technology Co., Ltd. (Wuhan, China). The Hydroxyproline ELISA Kit (cat. no. BWEM-928) was purchased from BIOSHARP (Beijing Lanjieke Technology Co., Ltd., Beijing, China). The Mouse IL-6 ELISA Kit (cat. no. SEKM-0007) was purchased from Beijing Solarbio Science & Technology Co., Ltd. (Beijing, China), and the Mouse IL-10 ELISA Kit (cat. no. BWEM-274) was purchased from BIOSHARP (Beijing Lanjieke Technology Co., Ltd., Beijing, China).

### 2.3. Equipment

The principal instruments used in this study included a refrigerated microcentrifuge (Heraeus Fresco 17; Thermo Fisher Scientific Inc., Waltham, MA, USA), an analytical balance (BSA124S-CW; Sartorius AG, Göttingen, Germany), a microplate reader (SpectraMax M2; Molecular Devices, LLC, San Jose, CA, USA), an automatic biochemical analyzer (Chemray 800; Rayto Life and Analytical Sciences Co., Ltd., Shenzhen, China), and an ultrapure water purification system (Aquaplore 3; Aquapro International Company LLC, Dover, DE, USA).

### 2.4. Animals and Experimental Design

**Figure 1:**
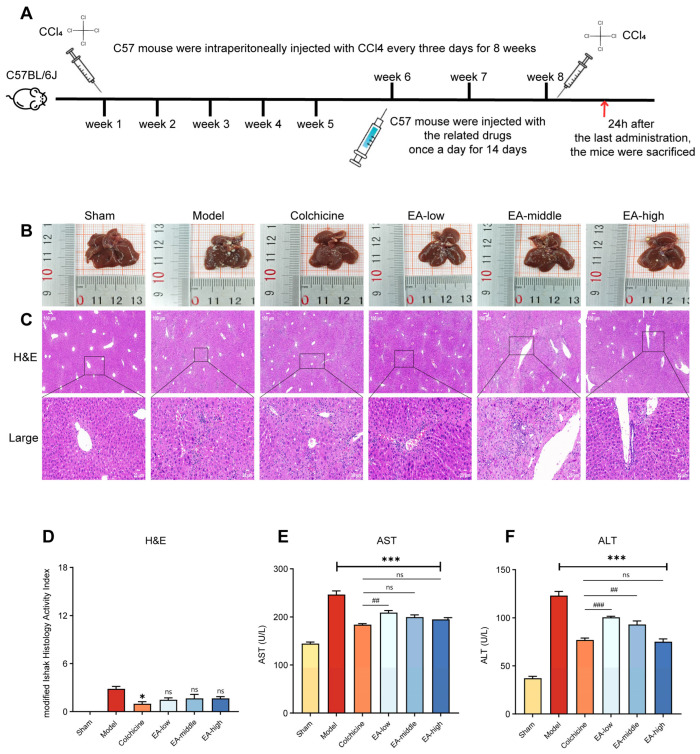
Effects of ellagic acid on liver morphology, histopathological injury, and serum aminotransferase activities in CCl_4_-treated mice. (**A**) Schematic illustration of the experimental design. Mice received intraperitoneal injections of CCl_4_ every 3 days for 8 weeks. Beginning in week 7, colchicine or ellagic acid (EA) was administered by oral gavage once daily for 14 consecutive days. (**B**) Representative images of gross liver morphology. (**C**) Representative H&E-stained liver sections. Boxed regions in the upper panels are presented at higher magnification in the lower panels. (**D**) Modified Ishak histological activity index. (**E**) Serum AST activity. (**F**) Serum ALT activity. Data are presented as mean ± SD (*n* = 7 per group). * *p* < 0.05 and *** *p* < 0.001 versus the model group; ## *p* < 0.01, and ### *p* < 0.001 versus the colchicine group; ns, not significant.

Forty-two specific-pathogen-free male C57BL/6J mice (8 weeks old; 20–22 g) were used in this study. All animal procedures were approved by the Animal Ethics Committee of Dali University (approval no. 2026PZ00059; approval date: 6 May 2026). Mice were randomly allocated to six groups (*n* = 7 per group): sham, model, colchicine, low-dose ellagic acid (EA-L, 30 mg/kg), medium-dose ellagic acid (EA-M, 60 mg/kg), and high-dose ellagic acid (EA-H, 120 mg/kg). Except for the sham group, hepatic fibrosis was induced by intraperitoneal administration of 20% CCl_4_ in olive oil (10 μL/g body weight) every 3 days for 8 weeks. Beginning in week 7 of CCl_4_ exposure, mice received colchicine (0.5 mg/kg) or EA at the indicated doses by oral gavage once daily for 14 consecutive days. Mice in the sham and model groups received an equivalent volume of saline by oral gavage. The experimental schedule is shown in Figure 1A.

### 2.5. Sample Collection

Following the final treatment administration, mice were fasted for 24 h and then euthanized. Blood samples were collected, and serum was separated by centrifugation at 3000 rpm for 15 min at 4 °C. Serum samples were stored at −80 °C until analysis. Liver tissues were excised, rinsed with cold saline, weighed, and photographed. The left hepatic lobe was fixed in 4% paraformaldehyde for histological examination. The remaining liver tissues were snap-frozen in liquid nitrogen and stored at −80 °C for subsequent biochemical and protein analyses.

### 2.6. Histopathological Analysis

Liver tissues were fixed in 4% paraformaldehyde, embedded in paraffin, and cut into 4-μm-thick sections. Sections were stained with hematoxylin and eosin (H&E) and Sirius Red according to standard procedures. Hepatic necroinflammatory activity was assessed in H&E-stained sections using the modified Ishak histological activity index (HAI). The total modified HAI score ranged from 0 to 18 and was calculated as the sum of four components: periportal or periseptal interface hepatitis (0–4), confluent necrosis (0–6), focal lytic necrosis, apoptosis, and focal inflammation (0–4), and portal inflammation (0–4).

Sirius Red-stained sections were used to evaluate collagen deposition. The Sirius Red-positive area was quantified as a percentage of the total tissue area using ImageJ software (version 1.8.0; National Institutes of Health, Bethesda, MD, USA). Histological assessments were independently performed by two observers blinded to group allocation, and discrepancies were resolved by consensus.

### 2.7. Biochemical and Inflammatory Assays

Serum alanine aminotransferase (ALT) and aspartate aminotransferase (AST) activities were measured using an automated biochemical analyzer. Hepatic malondialdehyde (MDA) and hydroxyproline (Hyp) contents and total superoxide dismutase (T-SOD) activity, as well as serum interleukin-6 (IL-6) and interleukin-10 (IL-10) concentrations, were determined according to the manufacturers’ instructions.

### 2.8. Immunohistochemistry

Paraffin-embedded liver sections underwent antigen retrieval, endogenous peroxidase blocking, and serum blocking before overnight incubation at 4 °C with primary antibodies against α-smooth muscle actin (α-SMA) and collagen type I alpha 1 chain (COL1A1). Sections were subsequently incubated with the corresponding horseradish peroxidase-conjugated secondary antibodies. Immunoreactivity was visualized using 3,3′-diaminobenzidine, and nuclei were counterstained with hematoxylin. Positive staining was quantified using ImageJ software.

### 2.9. Western Blot Analysis

Liver tissues were homogenized in radioimmunoprecipitation assay (RIPA) lysis buffer containing protease inhibitors. Supernatants containing total protein were collected after centrifugation. Protein concentrations were determined using a bicinchoninic acid (BCA) protein assay. Equal amounts of protein were separated by SDS-PAGE and transferred to polyvinylidene fluoride membranes. After blocking, the membranes were incubated overnight at 4 °C with primary antibodies against E-cadherin, N-cadherin, vimentin, AKT, PI3K, GAPDH, and β-actin and then with the corresponding horseradish peroxidase-conjugated secondary antibodies. Protein bands were visualized by enhanced chemiluminescence.

### 2.10. Network Pharmacology Analysis

Putative targets associated with EA, hepatic fibrosis, and epithelial–mesenchymal transition were retrieved from the TCMSP, GeneCards, SwissTargetPrediction, and OMIM databases (accessed on 14 October 2025). After duplicate targets were removed, overlapping targets were identified and visualized using a Venn diagram. Protein–protein interaction analysis was performed using the STRING database, and the resulting network was imported into Cytoscape version 3.9.1 for topological analysis and hub-target identification.

Gene Ontology and Kyoto Encyclopedia of Genes and Genomes pathway enrichment analyses were conducted using the DAVID database. A *p* value < 0.05 was considered statistically significant. The top enriched pathways were visualized using bubble plots.

### 2.11. Statistical Analysis

Data are presented as mean ± standard deviation (SD). Normality and homogeneity of variance were assessed using the Shapiro–Wilk test and Levene’s test, respectively. For data that satisfied both assumptions, differences among groups were analyzed using one-way analysis of variance followed by Tukey’s post hoc test. Otherwise, the Kruskal–Wallis test was applied. Statistical analyses were performed using GraphPad Prism version 9.5.1, and *p* < 0.05 was considered statistically significant.

## 3. Results

### 3.1. Ellagic Acid Attenuated CCl_4_-Induced Hepatic Injury in Mice

The hepatoprotective effects of ellagic acid (EA) were evaluated in mice with CCl_4_-induced hepatic injury. During weeks 7–8 of CCl_4_ administration, mice received colchicine or EA at three dose levels for 14 consecutive days (Figure 1A). Representative gross images showed that livers from the sham group had a smooth surface and normal appearance, whereas CCl_4_ exposure resulted in darker coloration and an irregular surface. These macroscopic abnormalities appeared less pronounced following colchicine or EA treatment, with the apparent improvement being greatest in the EA-H group (Figure 1B).

Histopathological examination was consistent with the gross observations. H&E-stained liver sections from the sham group showed preserved hepatic architecture. In contrast, the model group exhibited marked histopathological alterations, including disorganization of the hepatic lobular architecture, hepatocellular degeneration, and inflammatory cell infiltration. These changes appeared less prominent in the colchicine- and EA-treated groups, particularly in the EA-M and EA-H groups (Figure 1C).

The modified Ishak histological activity index was higher in the model group than in the sham group, consistent with successful establishment of CCl_4_-induced hepatic injury. Colchicine significantly reduced the histological activity score. Although the EA-treated groups showed lower mean scores than the model group, these differences did not reach statistical significance under the present experimental conditions (Figure 1D).

Serum aminotransferase activities were further assessed as biochemical indicators of hepatocellular injury. CCl_4_ administration markedly increased serum AST and ALT activities compared with those in the sham group. Treatment with colchicine or EA was associated with lower AST and ALT activities, with the greatest reductions observed in the EA-H group. Compared with the model group, EA-H significantly reduced serum AST and ALT activities (both *p* < 0.001; Figure 1E,F). Overall, these findings are consistent with a protective effect of EA against CCl_4_-induced hepatic injury, with the most consistent response observed at the highest tested dose.

### 3.2. Ellagic Acid Attenuated CCl_4_-Associated Fibrogenic Remodeling and Improved Oxidative Stress- and Inflammation-Related Indices in Mice

**Figure 2:**
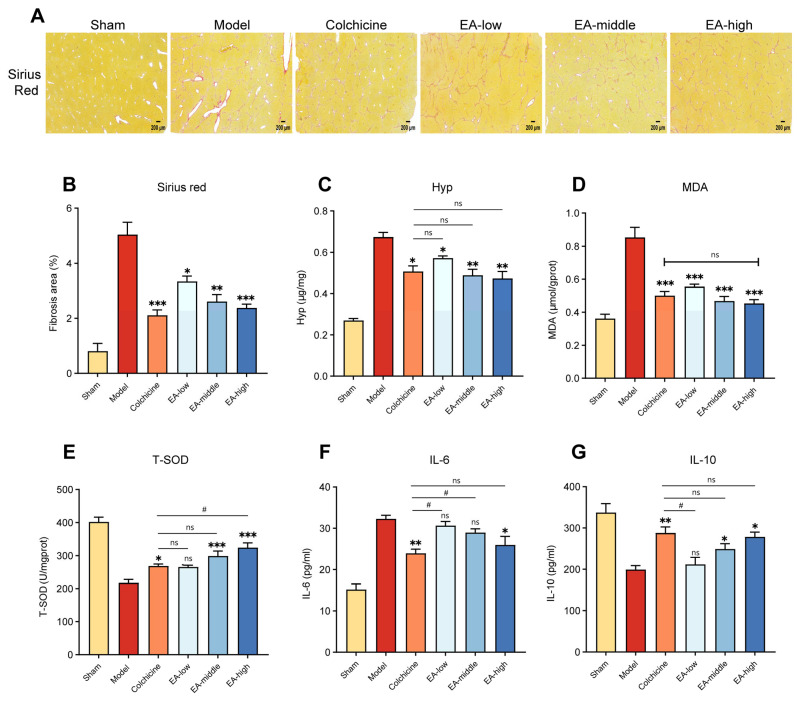
Effects of ellagic acid on collagen deposition and oxidative stress- and inflammation-related indices in CCl_4_-treated mice. (**A**) Representative Sirius Red-stained liver sections. (**B**) Quantification of the Sirius Red-positive area. (**C**) Hepatic hydroxyproline (Hyp) content. (**D**) Hepatic malondialdehyde (MDA) content. (**E**) Hepatic total superoxide dismutase (T-SOD) activity. (**F**) Serum interleukin-6 (IL-6) concentration. (**G**) Serum interleukin-10 (IL-10) concentration. Data are presented as mean ± SD (*n* = 7 per group). * *p* < 0.05, ** *p* < 0.01, and *** *p* < 0.001 versus the model group; # *p* < 0.05 versus the colchicine group; ns, not significant. Scale bar: 200 μm.

Fibrosis-associated extracellular matrix accumulation was further assessed. Sirius Red staining revealed extensive collagen deposition in the livers of CCl_4_-treated mice, whereas collagen staining appeared less extensive in the colchicine- and EA-treated groups (Figure 2A). Quantitative analysis showed that the Sirius Red-positive area was lower in the colchicine group and in all EA-treated groups than in the model group, with the greatest reduction among the EA-treated groups observed in the EA-H group (Figure 2B). Consistent with these histological findings, hepatic hydroxyproline (Hyp) content was significantly reduced in the colchicine, EA-L, EA-M, and EA-H groups (Figure 2C). These results indicate that EA attenuated CCl_4_-associated hepatic collagen accumulation.

Oxidative stress-related indices were then evaluated. Compared with the sham group, CCl_4_ exposure increased hepatic malondialdehyde (MDA) content and reduced total superoxide dismutase (T-SOD) activity. Hepatic MDA content was significantly lower in all treatment groups than in the model group (Figure 2D). In contrast, T-SOD activity was significantly increased in the colchicine, EA-M, and EA-H groups, whereas the increase in the EA-L group did not reach statistical significance (Figure 2E). Notably, T-SOD activity was higher in the EA-H group than in the colchicine group. This pattern was consistent with an improvement in hepatic antioxidant status following EA treatment.

Serum cytokine analyses showed that IL-6 concentrations were significantly lower in the colchicine and EA-H groups than in the model group, whereas the reductions observed in the EA-L and EA-M groups were not statistically significant (Figure 2F). Conversely, serum IL-10 concentrations were significantly increased in the EA-M and EA-H groups, whereas no significant difference was observed in the EA-L group (Figure 2G). IL-6 and IL-10 concentrations did not differ significantly between the EA-H and colchicine groups. Overall, EA treatment was associated with reduced collagen accumulation and improvements in oxidative stress- and inflammation-related indices, with the most consistent effects observed in the EA-H group.

### 3.3. Network Pharmacology Identified Candidate EA Targets Shared by Hepatic Fibrosis and EMT

**Figure 3:**
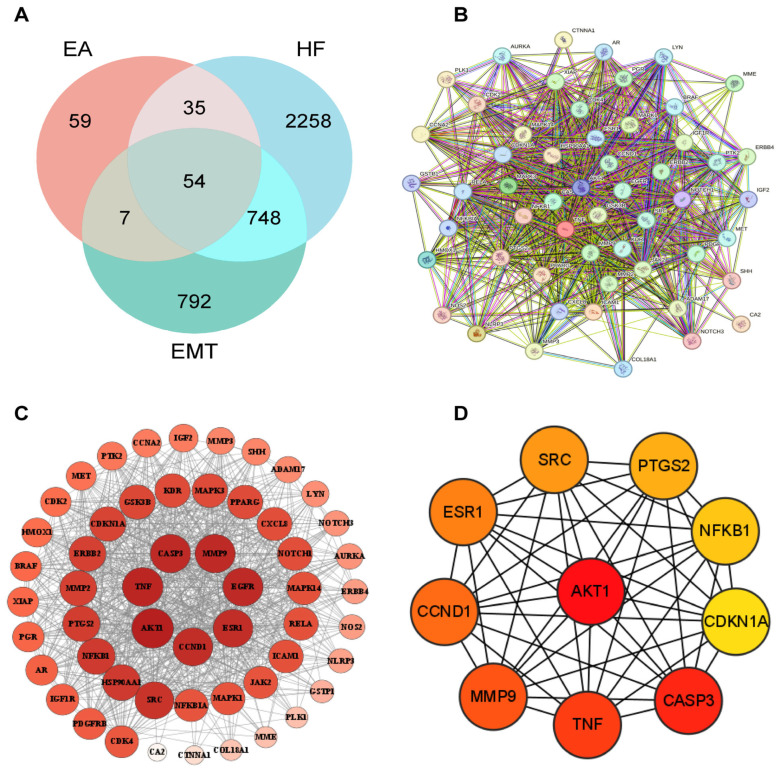
Target overlap and protein–protein interaction network analysis of ellagic acid, hepatic fibrosis, and epithelial–mesenchymal transition. (**A**) Venn diagram showing the overlap among putative ellagic acid (EA) targets, hepatic fibrosis (HF)-associated targets, and epithelial–mesenchymal transition (EMT)-associated targets. Fifty-four shared targets were identified. (**B**) PPI network of the 54 shared targets generated using the STRING database. (**C**) Cytoscape visualization of the PPI network. Node size and color intensity represent the relative topological importance of each target. (**D**) The 10 highest-ranked hub targets identified by topological analysis: AKT1, TNF, CASP3, MMP9, CCND1, ESR1, SRC, NFKB1, PTGS2, and CDKN1A.

Network pharmacology analysis was used to identify potential targets shared by EA, hepatic fibrosis (HF), and epithelial–mesenchymal transition (EMT). In total, 155 putative EA targets, 3095 HF-associated targets, and 1601 EMT-associated targets were retrieved from the selected databases. Venn diagram analysis identified 54 shared targets among EA, HF, and EMT (Figure 3A), suggesting that EA may be associated with HF through multiple targets relevant to EMT-associated pathological processes.

A protein–protein interaction (PPI) network was constructed to examine the relationships among the 54 shared targets. The network comprised 54 nodes and 816 edges, indicating extensive interactions among the identified targets (Figure 3B). Cytoscape-based visualization and topological analysis are shown in Figure 3C.

The 10 highest-ranked hub targets were AKT1, TNF, CASP3, MMP9, CCND1, ESR1, SRC, NFKB1, PTGS2, and CDKN1A (Figure 3D). These targets are associated with biological processes relevant to fibrogenesis, including cell survival, inflammatory responses, extracellular matrix remodeling, cell proliferation, and stress-related signaling. Among these targets, AKT1 was a highly connected hub in the PPI network, supporting the subsequent prioritization of PI3K/AKT-related signaling for enrichment analysis and experimental assessment.

### 3.4. Functional Enrichment Analysis Identified PI3K/AKT-Related Signaling as a Candidate Pathway

**Figure 4:**
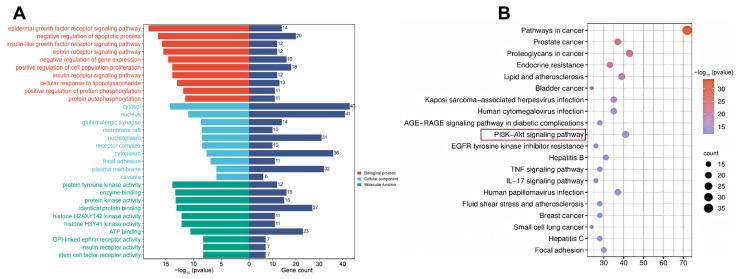
GO and KEGG enrichment profiles of targets shared by ellagic acid, hepatic fibrosis, and epithelial–mesenchymal transition. (**A**) GO enrichment analysis of the 54 shared targets. The top enriched terms in the biological process, cellular component, and molecular function categories are shown. The left and right bars indicate −log_10_(*p* value) and gene count, respectively. (**B**) KEGG pathway enrichment analysis of the shared targets. Bubble size represents gene count, whereas color represents −log_10_(*p* value). The highlighted PI3K/AKT signaling pathway was selected for subsequent experimental assessment.

The functional relevance of the 54 shared targets was assessed by Gene Ontology (GO) and Kyoto Encyclopedia of Genes and Genomes (KEGG) enrichment analyses. A total of 649 GO terms met the significance threshold. Enriched biological process terms were associated with epidermal growth factor receptor signaling, regulation of apoptotic processes, protein phosphorylation, and cell proliferation (Figure 4A). The enriched cellular component terms included the cytosol, nucleus, plasma membrane, membrane rafts, and focal adhesions. In the molecular function category, the shared targets were primarily enriched in protein tyrosine kinase activity, protein kinase activity, enzyme binding, and protein binding.

KEGG enrichment analysis identified 136 significantly enriched pathways. In addition to cancer- and cellular stress-related pathways, several pathways relevant to fibrogenesis, inflammatory responses, and cell-survival signaling were enriched, including the PI3K/AKT, TNF, and IL-17 signaling pathways (Figure 4B). PI3K/AKT-related signaling was selected for subsequent experimental assessment because the pathway was enriched among the shared targets and AKT1 was identified as a highly connected hub target in the protein–protein interaction network. Given the established involvement of PI3K/AKT signaling in hepatic stellate cell activation, cell survival, and EMT-associated molecular changes, these results provided a network-based rationale for evaluating its association with the protective effects of EA in CCl_4_-induced hepatic fibrosis.

### 3.5. High-Dose EA Attenuated Fibrosis- and EMT-Associated Protein Alterations and Reduced PI3K and AKT Protein Abundance

Markers of hepatic stellate cell activation, extracellular matrix accumulation, EMT-associated protein alterations, and PI3K/AKT-related signaling were assessed in liver tissues from the sham, model, and EA-H groups.

**Figure 5:**
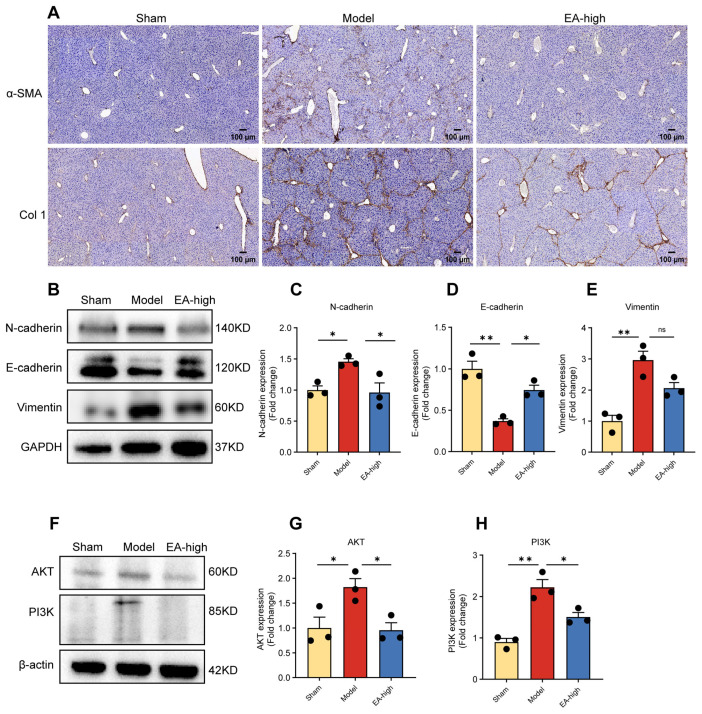
Effects of EA-H on fibrosis-associated, EMT-associated, and PI3K/AKT-related protein alterations in CCl_4_-treated mouse livers. (**A**) Representative immunohistochemical staining for α-SMA and collagen I (COL1A1) in liver sections from the sham, model, and EA-H groups. Scale bar: 100 μm. (**B**) Representative Western blots of N-cadherin, E-cadherin, Vimentin, and GAPDH. (**C**–**E**) Densitometric analyses of N-cadherin, E-cadherin, and Vimentin expression, respectively. (**F**) Representative Western blots of AKT, PI3K, and β-actin. (**G**,**H**) Densitometric analyses of AKT and PI3K expression, respectively. Data are presented as mean ± SD (*n* = 3 biological replicates per group for panels **C**–**E**,**G**,**H**). Statistical comparisons are indicated by horizontal bars. * p < 0.05; ** p < 0.01; ns, not significant.

Immunohistochemical staining showed low immunoreactivity for α-SMA and collagen I (COL1A1) in the sham group. In contrast, CCl_4_ exposure was associated with markedly increased immunoreactivity for both proteins in the model group, consistent with enhanced hepatic stellate cell activation and collagen I accumulation. Compared with the model group, the EA-H group showed lower α-SMA and COL1A1 immunoreactivity (Figure 5A).

Western blot analysis showed that CCl_4_ treatment increased N-cadherin expression and decreased E-cadherin expression relative to the sham group. EA-H significantly reduced N-cadherin expression and increased E-cadherin expression compared with the model group (both *p* < 0.05; Figure 5B–D). Vimentin expression was significantly elevated in the model group; although its mean expression was lower in the EA-H group, the difference did not reach statistical significance (Figure 5B,E).

Total AKT and PI3K protein abundance were significantly higher in the model group than in the sham group. EA-H significantly reduced the abundance of both proteins relative to the model group (Figure 5F–H). These findings indicate that EA-H was associated with attenuation of fibrosis- and EMT-associated protein alterations in CCl_4_-treated livers, together with reduced PI3K and AKT protein abundance.

## 4. Discussion

In this CCl_4_-induced mouse model of hepatic fibrosis, EA administration was associated with improvements in gross liver appearance and histopathological findings, lower collagen deposition and hepatic hydroxyproline content, and reduced serum ALT and AST activities. Hepatic malondialdehyde content was lower, whereas total superoxide dismutase activity was higher. In parallel, serum interleukin-6 concentrations decreased, and interleukin-10 concentrations increased, with the most consistent effects observed in the EA-H group. In the EA-H group, lower α-SMA and collagen I (COL1A1) immunoreactivity was accompanied by reduced N-cadherin, PI3K, and AKT protein abundance and increased E-cadherin protein abundance. Although vimentin expression showed a downward trend relative to the model group, the difference was not statistically significant. Collectively, these findings indicate that EA attenuated CCl_4_-induced hepatic fibrosis and was associated with improved oxidative stress- and inflammation-related indices, lower expression of markers related to HSC activation and collagen deposition, and partial normalization of EMT-associated protein expression.

These observations are consistent with previous evidence supporting the hepatoprotective and anti-fibrotic potential of EA. In a high-fat and high-fructose diet-induced mouse model of NAFLD, EA reduced lipid accumulation, insulin resistance, oxidative stress, and inflammation. The beneficial effects were more pronounced in mice with a high urolithin A-producing capacity [9]. In a model of iron overload, EA attenuated hepatic injury and fibrosis and was associated with improved iron metabolism and reduced ferroptosis through modulation of TGF-β/Smad signaling [10]. In addition, EA derived from whole pomegranate fruit reduced liver injury in a rat model of hepatic cholestasis [11]. Mechanistic evidence has also indicated that EA may limit fibrotic progression by inducing ferroportin-dependent ferroptosis and impairing SNARE complex formation in activated HSCs [12]. Consistent with these experimental findings, a randomized double-blind clinical trial reported that EA supplementation improved some liver enzyme and oxidative stress-related markers in patients with NAFLD [13]. The effects observed in the present CCl_4_-induced model therefore complement previous evidence by demonstrating that EA was associated with improvements in histopathological injury, biochemical indices, and fibrosis-related outcomes.

Persistent activation of hepatic stellate cells and excessive extracellular matrix deposition are central features of hepatic fibrogenesis. PI3K/AKT/mTOR signaling has been implicated in HSC activation and fibrogenic remodeling in experimental models of hepatic fibrosis [14]. Against this background, the EA-H group showed lower total PI3K and AKT protein abundance, increased E-cadherin expression, and reduced N-cadherin expression. Vimentin expression also tended to be lower than that in the model group, although the difference was not statistically significant. These findings indicate that EA treatment was associated with changes in PI3K/AKT-related proteins and partial normalization of EMT-associated protein expression. However, the phosphorylation status of PI3K and AKT was not assessed. Therefore, the present data do not demonstrate direct inhibition of PI3K/AKT pathway activation. Instead, they support an association between EA treatment, reduced total PI3K and AKT protein abundance, and changes in EMT-associated protein expression. Further studies incorporating phospho-PI3K and phospho-AKT measurements, together with pathway-specific pharmacological or genetic approaches, are required to determine whether PI3K/AKT signaling directly contributes to the anti-fibrotic effects of EA.

Colchicine was included as a pharmacological positive control because of its established anti-inflammatory activity [15]. Direct comparisons between the EA-H and colchicine groups showed no statistically significant differences in serum ALT or AST activities, hepatic Hyp or MDA content, or serum IL-6 or IL-10 concentrations. In contrast, hepatic T-SOD activity was significantly higher in the EA-H group than in the colchicine group. Colchicine significantly reduced the modified Ishak histological activity score, whereas the reduction in the EA-H group did not reach statistical significance. Thus, the relative effects of EA-H and colchicine varied across the evaluated endpoints. As the study was not designed as an equivalence or non-inferiority trial, nonsignificant differences between EA-H and colchicine cannot be interpreted as evidence of comparable efficacy.

Available clinical evidence for EA in liver disease is derived mainly from studies in metabolic liver disease rather than established hepatic fibrosis. In a randomized, double-blind trial in patients with non-alcoholic fatty liver disease, EA supplementation was reported to improve inflammatory markers and adiponectin levels [16]. EA has also been evaluated in a randomized, add-on, double-blind, controlled trial in patients with metabolic dysfunction-associated steatotic liver disease [17]. However, these studies do not establish whether EA is effective in patients with established hepatic fibrosis. Differences in patient populations, disease stage, treatment duration, and outcome measures also preclude direct comparison with the present CCl_4_-induced fibrosis model. The clinical effects of EA may also vary according to the formulation used and individual host metabolism. Free EA and pomegranate juice differ in bioavailability and bioactivity [18]; therefore, findings obtained with one preparation should not be generalized to another. EA and ellagitannins are converted to urolithins by human fecal microbiota, which may further affect systemic exposure and biological response [19]. Human studies have also identified distinct urolithin metabotypes, indicating substantial interindividual variation in the metabolism of ellagitannin- and EA-containing foods [20]. Whether similar microbiota-dependent variability occurs in human liver disease, particularly hepatic fibrosis, remains unknown. Future clinical trials of EA in hepatic fibrosis should clearly define the formulation used, include pharmacokinetic and microbial-metabolite assessments, and use fibrosis-relevant endpoints with sufficient follow-up to evaluate efficacy and safety. The present findings provide preclinical support for such studies but do not establish clinical efficacy.

Further evaluation of EA should address not only treatment efficacy but also factors that may influence systemic exposure and biological response. Strategies to improve the solubility, stability, and bioavailability of EA warrant investigation. Future studies should also distinguish the effects attributable to free EA, ellagitannin-rich preparations, and microbiota-derived urolithins. In hepatic fibrosis, the present study provides preclinical evidence across histopathological, biochemical, and molecular endpoints, whereas the available clinical evidence remains limited to small randomized clinical trials in patients with NAFLD, including a trial of purified EA [16] and a randomized trial of pomegranate extract [21]. Appropriately powered randomized controlled trials in patients with liver fibrosis, supported by pharmacokinetic assessments and longer follow-up, are needed to determine the efficacy and safety of EA-based interventions.

This study has several limitations. First, the anti-fibrotic effects of EA were evaluated in a single CCl_4_-induced mouse model. Although this model is widely used, it does not reflect the etiological diversity or full pathological complexity of human hepatic fibrosis. Second, only total PI3K and AKT protein abundance were measured. Because AKT activation is regulated by phosphorylation at key residues, including Thr308 and Ser473 [22], reduced total PI3K and AKT protein abundance does not establish inhibition of PI3K/AKT pathway activation. The present data therefore support an association between EA treatment and changes in PI3K/AKT-related protein abundance, rather than direct inactivation of the PI3K/AKT pathway. Future studies should assess phosphorylated AKT at Thr308 and Ser473, together with phosphorylation changes in upstream PI3K-related signaling components, and incorporate pathway-specific pharmacological or genetic approaches. Third, the assessment of EMT-associated changes was limited to E-cadherin, N-cadherin, and vimentin. These findings support partial normalization of EMT-associated protein expression but do not establish EMT as a direct mediator of the anti-fibrotic effects of EA. Key transcriptional regulators, including Snail, Slug, Twist, and ZEB1, were not examined. Cell type-specific analyses and functional experiments will be needed to define the contribution of EMT-associated changes to the observed anti-fibrotic response. Finally, network pharmacology identified several candidate targets and pathways, including TNF, NFKB1, MMP9, and CASP3, whereas experimental validation was limited to PI3K/AKT-related proteins. PI3K/AKT-related signaling was selected because it was enriched in the KEGG analysis and AKT1 ranked among the central nodes in the protein–protein interaction network. The network pharmacology results should therefore be regarded as hypothesis-generating, and the present experiments represent focused validation of one candidate pathway rather than confirmation of the full predicted regulatory network. Additional studies should examine TNF/NF-κB signaling, MMP9, CASP3, and other predicted targets to clarify the potential multi-target actions of EA in hepatic fibrosis.

## 5. Conclusions

EA attenuated CCl_4_-induced hepatic fibrosis in mice and was associated with reduced oxidative stress and inflammation, lower expression of markers related to HSC activation and collagen deposition, and partial normalization of EMT-associated protein expression. These effects were accompanied by reduced total PI3K and AKT protein abundance. However, the present findings do not establish direct inhibition of PI3K/AKT pathway activation. Further studies are needed to assess the pharmacokinetics, mechanisms of action, and clinical potential of EA in hepatic fibrosis.

## Data Availability

The original contributions presented in this study are included in the article. Further inquiries can be directed to the corresponding author.
